# Delphi method applicability in drug foresight

**DOI:** 10.1186/s13011-024-00617-7

**Published:** 2024-07-27

**Authors:** Tomi Lintonen, Karoliina Karjalainen, Sanna Rönkä, Elina Kotovirta, Solja Niemelä

**Affiliations:** 1 Finnish Foundation for Alcohol Studies, PO Box 30, Helsinki, FI-00271 Finland; 2 Finnish Institute for Health and Welfare (THL), Helsinki, Finland; 3 Ministry of Social and Health Affairs, Helsinki, Finland; 4 Department of Psychiatry, University of Turku, Turku, Finland; 5 Department of Psychiatry, Addiction Psychiatry Unit, Turku University Hospital, The Wellbeing Services County of Southwest Finland, Turku, Finland

**Keywords:** Foresight, Delphi, Drugs, Evaluation, Applicability

## Abstract

**Background:**

The aim of the current study was to assess the accuracy of expert predictions, which were derived using a Delphi panel foresight study between 2009 and 2011, on a variety of drug-related topics in Finland in 2020.

**Methods:**

The material used to evaluate the accuracy of the predictions consists of published reports on statistics, survey results, official register data, wastewater analyses and official documents. Whenever possible, we used multiple information sources to ascertain possible changes related to the predictions.

**Results:**

Between 2009 and 2011, the majority – but not all – of the experts accurately predicted an increase in drug use. Indeed, more people experimented with or used drugs, and more drug residues were found in wastewater monitoring. The experts also correctly predicted an increase in population-level approval of drug use, but this development has been rather slow. Contrary to predictions, there was no marked increase in the use of new synthetic drugs. However, the misuse of buprenorphine increased during the 2010s. In the drug market, unit prices were surprisingly stable over the ten-year period. There were no changes in legislation related to the legal status of drugs, as was foreseen by the experts. However, enforcement moved in the direction foreseen by the experts: more lenient measures have been taken against users. Drug care system reforms favored a combination of mental health and addiction care units between 2009 and 2011, and 2020, as foreseen by the experts.

**Conclusions:**

It seems to have been easier for the experts to foresee the continuation of existing trends, e.g., increasing use of drugs or widening approval of drugs, than to predict possible changes in the popularity of distinct groups of drugs such as new psychoactive substances (NPS). Even armed with the prediction that drug imports and wholesale would increasingly fall into the domain of organized crime, this undesirable development could not be stopped. Expert disagreement can also be seen as a valuable indication of uncertainty regarding the future. Foresight related to drug-related issues can produce relatively accurate and realistic views of the future at least up to ten years ahead.

## Background

Foresight is an umbrella term used to describe methods for increasing knowledge about the future and applying this knowledge in decision-making. The field of foresight emerged in the 1950s and has been progressing into organizational integration since the turn of the millennium [1, 2]. This progress has manifested as an increase in scientific literature utilizing foresight methods [3] as well as organizational statements regarding the role of foresight [4]. Initially, foresight was strongly associated with science, technology and innovation (STI) policy-making, but its scope has expanded into broader strategic issues in the private sector. Recently, the use of foresight has also increased in many areas of public interest, such as the field of licit and illicit drugs. In the first decade of the current millennium, drug foresight was in an experimental phase. In Great Britain, emanating from the STI field, UK Drugs Foresight made predictions about the drug situation in twenty years [5]. This timeframe was deemed very ambitious; the Finnish drugs foresight exercise settled for a more modest ten-year scope [6].

Drug foresight exercises often publish their reports as working papers (e.g., [7]), as this practice is useful for individuals who need the information to prepare for and shape the future. Some peer-reviewed papers have also been published in recent years. Continuing the STI tradition, the number of foresight studies on pharmaceutical drugs has been increasing (e.g., [8]). As part of an academic initiative supported by the European Monitoring Centre for Drugs and Drug Addiction (EMCDDA) to “…speculate on what drugs and related intervention and policy might become…”, several papers [9–12] were published together, and one of the papers drew conclusions on the use of foresight in the field of drugs [13]. However, to our knowledge, no peer-reviewed papers have provided a retrospective analysis of foresight predictions, i.e., looking back at how well the predictions lined up with actual developments.

The aim of the current study was to assess the accuracy of expert predictions, which were derived using a Delphi panel study between 2009 and 2011, on a variety of drug-related topics in Finland in 2020 [6, 14]. In addition to checking the status in predicted areas of the drug situation, this study aims to analyze and draw conclusions on factors affecting the accuracy of predictions. Furthermore, the paper will discuss implications for drug situation surveillance as well as implications for foresight exercises in the drug field.

## Methods

### The Delphi study conducted between 2009 and 2011

Experts’ predictions were collected between 2009 and 2011 using a Real Time Delphi [15] online service and published in 2014 [6]. The European Monitoring Centre for Drugs and Drug Addiction (EMCDDA) coordinates a network of national focal points (NFPs) set up in the 27 EU Member States, Norway and Türkiye. The network is called Reitox, the European Information Network on Drugs and Drug Addiction. In Finland, the focal point was established in 1995 and the members of the national network of experts were used as informants in this Delphi study. The number of experts was 43 at the start of the study process. They have been assigned to this network to provide the European Union with comprehensive information on the drug situation in Finland. The experts came mainly from the public sector from institutions like the Finnish Institute for Health and Welfare, Statistics Finland, University of Helsinki, Ministry of Justice, Ministry of Social Affairs and Health, Ministry of Education and Culture, Finnish Customs, National Bureau of Investigation (Police), City of Helsinki, the Nordic Welfare Centre, Finnish Medicines Agency, but relevant third sector actors were also involved such as the SOSTE Finnish Federation for Social Affairs and Health, the Helsinki Deaconess Institute and the A-Clinic Foundation. These institutions and organizations represent sectors involved in drug monitoring and policy planning in Finland. The experts are professionals in e.g. social sciences, medicine, chemistry, police, civil service, and law, and were in a position to estimate the development of Finnish drug situation.

The invitations to take part in the discussions were sent to the Finnish Reitox experts email list. Taking part was voluntary and absolutely anonymous; not even the researchers could know who answered what. This was done to encourage honest personal views instead of so called official institutional views. Three Delphi rounds were conducted online during 2009–2011. The number of experts taking part in each of the three Delphi discussion rounds was 19, 18 and nine. The time for each discussion was limited to two weeks per round. Themes for the first Delphi round were developed within the research team. The second and third rounds included questions and topics formulated on ideas presented by the experts during the preceding rounds. Certain statements were re-issued on successive rounds, both in same wording and in slightly developed forms. This was done to clarify the issues in terms of evaluations of probability and justifications.

In contrast with survey questionnaires, the web-based response platform was open for the experts to view others’ responses and adjust their own during the two-week window for each discussion round. The Delphi method aims at promoting deliberation and making informed predictions on the future, and encourages information sharing. The experts were able to view the preliminary results, both quantitative and qualitative. They were also able to amend or change their own views in case other justifications made sense to them.

### The evaluation study in 2023

In the present study, the material used to evaluate the accuracy of the predictions consists of published reports on statistics, survey results, official register data, wastewater analyses and official documents. Most of these documents have been produced due to national interest and published in the Finnish language. However, as the documents are available online, interested readers can use automated translation services to ascertain the facts and study specific issues further. Whenever possible, we used multiple information sources to ascertain possible changes related to the predictions.

The “Drug use and use patterns” section draws mainly on reports published by the Finnish Institute for Health and Welfare (THL) and Statistics Finland public databases. In addition, personal communication from a staff member of the Finnish Institute for Health and Welfare was used to evaluate postmortem toxicology findings of new synthetic drugs – this was done since no data on the issue were available publicly. In the nationally representative survey series on “Drug use and drug attitudes among Finns – Drug-related population surveys in Finland 1992–2018” [16], the respondents were aged between 15 and 69 years and randomly sampled from the Finnish population register. In 2010, the response rate was 48% (*N* = 2023); in 2014, it was 50% (*N* = 3485); and in 2018, it was 46% (*N* = 3229). The data were collected by Statistics Finland, and the structure and questions in the surveys were similar, thus enabling us to analyze population trends. The “Drug situation in Finland 2020” [17] continues the tradition of national reports that draw upon a variety of data sources. Data on illicit drug use among nonmedical users of medicine were published by Karjalainen et al. [18] and amended using the most recent data published on the web pages of the Finnish Institute for Health and Welfare.

Data on “Drug markets” were derived from the EMCDDA public statistics [19] on drug retail prices; in the case of Finland, these data were initially produced by the National Bureau of Investigation (NBI; Police of Finland). Reports by the Finnish Institute for Health and Welfare 2020 [20] and the Criminal Sanctions Agency (Finland) [21] documents were quoted.

In the area of “Drug control”, the National Police Board of Finland and National Prosecution Authority documents were utilized as well as police statistics data. Population surveys on public attitudes toward punishments were also reported by Karjalainen et al. [16]. When evaluating the trends in new psychoactive substances, data from the Finnish Customs Laboratory were used. Drug law experts within the Ministry of Social Affairs and Health were consulted for possible revisions of drug laws.

The analyses on possible changes in “Health, care and the care system” used survey data published in the report entitled “Drug situation in Finland 2020” [17]. Data from the Finnish National Infectious Diseases Register were used to assess trends in human immunodeficiency virus (HIV) [22]. A recent peer-reviewed article on opioid addiction treatment published information on time trends in substitution treatment [23]. The statistical yearbook of alcohol and drug statistics 2021 [20] and a THL report on drug care services [24] were utilized to assess the developments in care services in the 2010s. The issue of drug consumption rooms was recently analyzed in a peer-reviewed article [25] after which a citizens’ initiative [26] was made; the initiative gained enough (over 50 000) signatures to warrant parliamentary processing. The Ministry of Social Affairs and Health was contacted to gather information on possible legislative processes underway, e.g., involuntary care of people who use drugs.

The predictions under “Attitudes toward drugs” were assessed using nationally representative survey data [16], a citizens’ initiative and news media.

## Results

### Drug use and use patterns

As predicted in the study between 2009 and 2011, surveys, register-based information, and wastewater analyses indicate that the use of cannabis, amphetamine and other illegal drugs has become more common from 2010 to 2020 (Table 1). The use of drugs has, in fact, increased even among older adults. In addition, population aging has been slow during this period. Contrary to predictions, the use of new synthetic drugs did not become more common from 2010 to 2018. Prescription drug abuse did not change considerably except for opioid use, which increased in the 2010s and consisted almost only of nonmedical use of opioid pharmaceuticals, most commonly buprenorphine but also oxycodone, methadone, tramadol and fentanyl, while heroin use is extremely rare in Finland [27]. As predicted, divergence of drug use cultures has not taken place: simultaneous use of cannabis and alcohol has increased slightly, and illicit drug use among nonmedical users of medicine has become more common (Table 1).

**Table 1:**
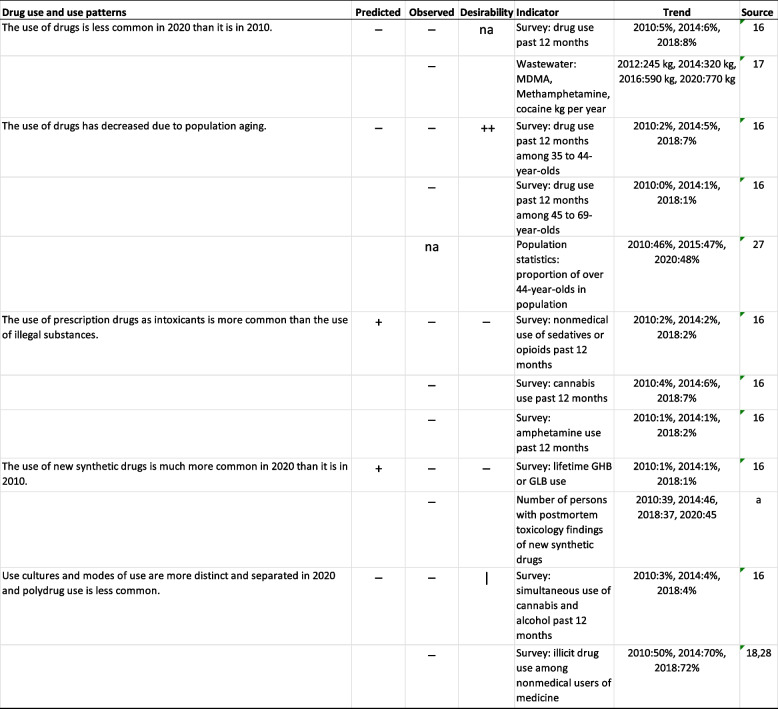
Trends in drug use and use patterns from 2009-2011 to 2020. Symbols

**Predicted**: "--" no-one agreed, "-" more than two thirds disagreed, "+" more than 2/3 agreed, "++" everyone agreed. **Observed**: “-“ stated change did not come true, “+” stated change materialized, “|” ambiguous, “na” not applicable. **Desirability**: “--" no-one held desirable, "-" more than two thirds held undesirable, "+" more than 2/3 held desirable, "++" everyone held desirable, “na” not applicable. **Source**: a Pirkko Kriikku (Finnish Institute for Health and Welfare) personal communication February 2022 [28, 29]

### Drug markets

The experts were undecided on the prediction that “Drug prices decrease due to supply exceeding demand” (Table 2), but no significant trends were seen in drug prices during the ten-year period. The experts did not believe that “Drugs and prescription medicine partly replaced alcohol drinking”. At the population level, there was a decrease in total alcohol consumption (Table 2), and the prevalence of drug use in the past 12 months increased from 2010 to 2018 (Table 1). Nonmedical use of sedatives or opioids in the past 12 months remained unchanged during the same period (Table 1). At the individual level, the simultaneous use of both cannabis + alcohol and prescription medicine + alcohol in the past 12 months increased slightly from 2010 to 2018 (Table 1). However, individual-level studies have shown that increased drug and prescription medicine have not replaced alcohol but have been combined with alcohol use [30, 31].

**Table 2:**
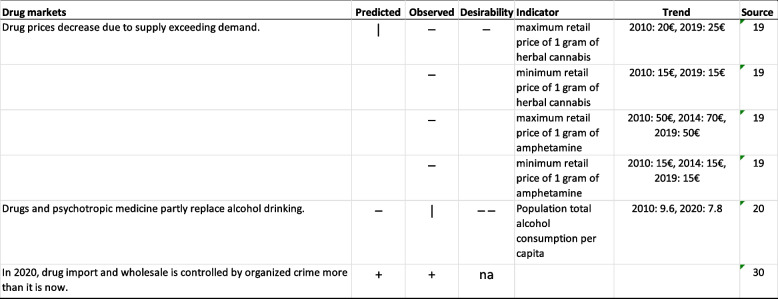
Trends in drug markets from 2009-2011 to 2020

**Predicted**: "--" no-one agreed, "-" more than two thirds disagreed, "+" more than 2/3 agreed, "++" everyone agreed. **Observed**: “-“ stated change did not come true, “+” stated change materialized, “|” ambiguous, “na” not applicable. **Desirability**: “--" no-one held desirable, "-" more than two thirds held undesirable, "+" more than 2/3 held desirable, "++" everyone held desirable, “na” not applicable

Finally, it was predicted that “Drug import and wholesale is controlled by organized crime more [in 2020] than it is [between years 2009 and 2011]”. The National Bureau of Investigations reported that while professional crime used to be loosely organized in Finland, the degree of discipline and hierarchy increased during the period [32]. This was deduced from, e.g., drug crime cases increasing in size. In particular, criminal motorcycle gangs have a strong hold of domestic markets and have tight networks abroad. This picture is supported by statistics from the Criminal Sanctions Agency: the number and proportion of prisoners belonging to organized criminal groups increased rapidly from 2010 to 2019 [21].

### Drug control

In line with the experts’ predictions, “drug use and possession for own use” was still illegal in 2020 (Table 3). The experts were, however, undecided in the study conducted between 2009 and 2011 regarding the illegality of use and possession of cannabis in 2020; no change in legislation was seen (Table 3). Nonetheless, a directive given by the National Police Board of Finland [33] now states that the Police can refrain from action against a user if she or he has sought treatment approved by the Ministry of Social Affairs and Health. This was made possible by a amendment to the law in 2014; previously adherence to treatment was required. As a result, the yearly numbers of treatment counseling notifications increased dramatically (Table 3). Since 2019, a drug crime case can also be closed by giving the person a notice (Table 3). The experts

**Table 3:**
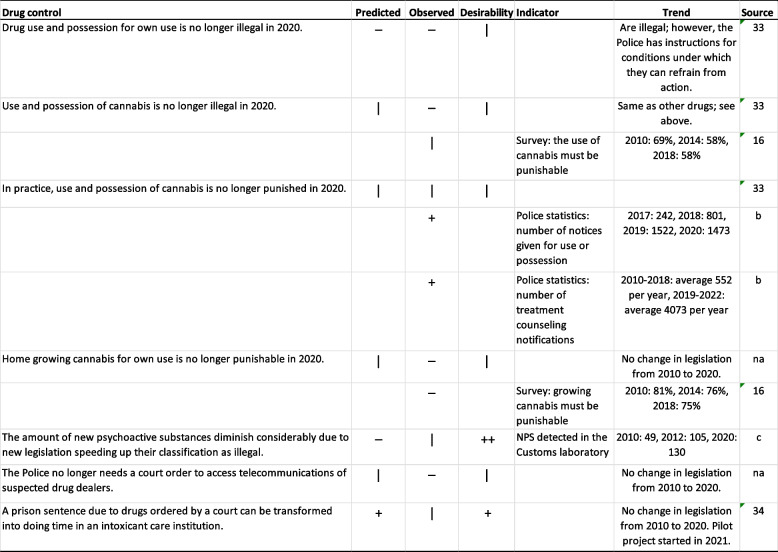
Trends in drug control from 2009-2011 to 2020

**Predicted**: "--" no-one agreed, "-" more than two thirds disagreed, "+" more than 2/3 agreed, "++" everyone agreed. **Observed**: “-“ stated change did not come true, “+” stated change materialized, “|” ambiguous, “na” not applicable. **Desirability**: “--" no-one held desirable, "-" more than two thirds held undesirable, "+" more than 2/3 held desirable, "++" everyone held desirable, “na” not applicable.  **Source: b** Juha Helenius, Police Statistics (Police College of Finland) personal communication February 2022.  **Source: c** Katja Pihlainen (Finnish Medicines Agency) personal communication February 2022

were undecided on the prediction that “In practice, use and possession of cannabis is no longer punished in 2020”; looking at the data presented in Table 3, it seems that the police have gradually shifted their directives and practices toward more lenient forms of punishment. Likewise, population surveys show that public support for punishments for the use and possession of cannabis has declined (Table 3).

The experts did not agree on the prediction that “Home growing cannabis for personal use will no longer be punishable in 2020”; in 2020, home growing was still illegal and punishable in Finland. Public support for illegality also remained strong (Table 3). “The amount of designer drugs will diminish considerably due to new legislation speeding up their classification as illegal” was viewed as unlikely by the experts in the Delphi study between 2009 and 2011. When the experts made their prediction, the yearly number of NPS detected in the Finnish Customs Laboratory was rapidly increasing. In 2011, the scope of the Narcotics Act was expanded through an amendment, and new psychoactive substances could be brought under legislative control based on a national harm assessment process [34], after which the yearly numbers fluctuated with no clear trend (Table 3). As predicted, the number of NPS did not decrease due to the new legislation; however, it seems likely that legislation amendment halted the rapid increase and led to better control of the NPS situation.

In the study conducted between 2009 and 2011, the experts were not unanimous regarding the statement “The Police no longer needs a court order to access telecommunications of suspected drug dealers”. No moves in this direction were made as of 2020 (Table 3). The development toward “A prison sentence due to drugs ordered by a court can be transformed into doing time in an intoxicant care institution” was viewed both as desirable and likely by the experts. In 2021, the prison service started a project to place prisoners with short prison sentences and substance abuse problems in an out-of-prison substance abuse treatment facility [35]. Initiatives to change the laws have not been proposed.

### Health, care and the care system

Table 4 shows that the experts were correct in predicting that no serious HIV epidemics would be experienced among users of injectable drugs by the year 2020: the incidence of HIV per year has slowly decreased. The experts correctly predicted that the use of prescription drugs as intoxicants would cause more deaths in 2020 than in 2009–2011. The most common cause of death in forensic investigations was buprenorphine; heroin was rarely reported as the cause of death (Table 4). Overall, the number of individuals who died due to prescription drugs increased by 23% from 2013 to 2019 (from 352 to 433). During the same period, the number of deaths due to buprenorphine increased by 37%. Therefore, the role of prescription drugs in drug-related deaths strengthened during this period.

**Table 4:**
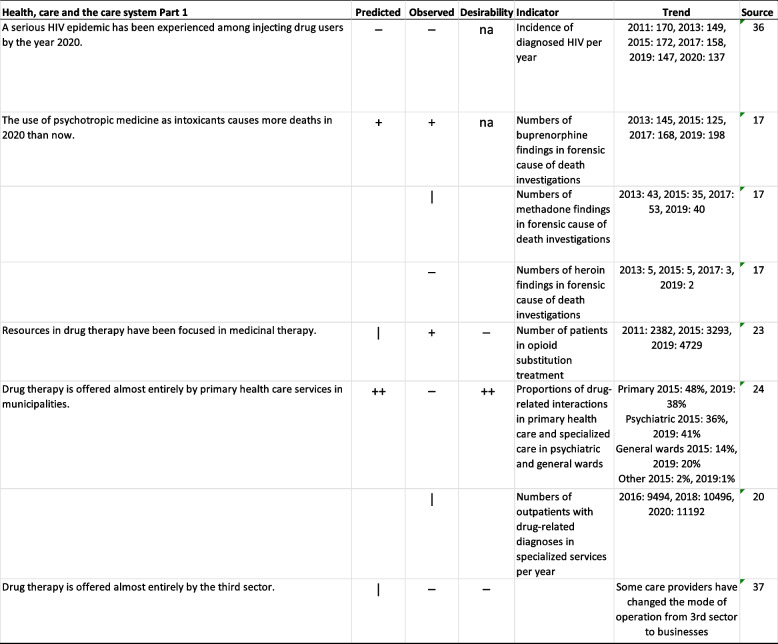
Predicted health, care and care system changes from 2009-2011 to 2020, Part 1

**Predicted**: "--" no-one agreed, "-" more than two thirds disagreed, "+" more than 2/3 agreed, "++" everyone agreed. **Observed**: “-“ stated change did not come true, “+” stated change materialized, “|” ambiguous, “na” not applicable. **Desirability**: “--" no-one held desirable, "-" more than two thirds held undesirable, "+" more than 2/3 held desirable, "++" everyone held desirable, “na” not applicable [36, 37]

The experts did not agree or disagree with the statement “Resources in drug therapy have been focused on medicinal therapy” in 2009–2011. Drug therapy seems to have shifted in that direction, as shown in Table 4: the number of patients in opioid substitution treatment increased more rapidly than the number of outpatients with drug-related diagnoses in specialized services (Table 4).

The statement “Drug therapy is offered almost entirely by primary health care services in municipalities” was widely agreed upon in the study conducted between 2009 and 2011; however, both statistics and two specific data collections on service use show increased utilization of specialized health care (Table 4). For the statement “Drug therapy is offered almost entirely by the third sector”, which left the experts undecided in the study conducted between 2009 and 2011 (Table 5), the third sector drug actors have, in fact, changed the mode of operation of their care services to businesses (e.g., 37).

**Table 5:**
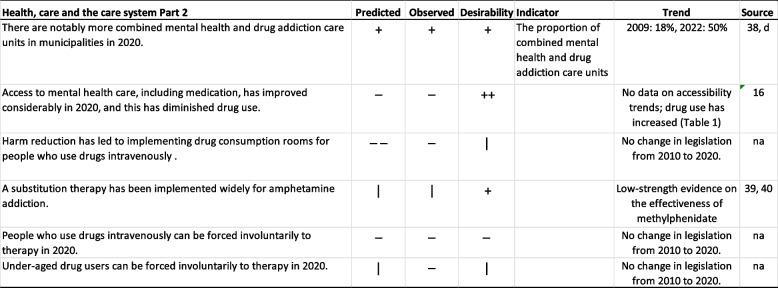
Predicted health, care and care system changes from 2009-2011 to 2020, Part 2

**Predicted**: "--" no-one agreed, "-" more than two thirds disagreed, "+" more than 2/3 agreed, "++" everyone agreed. **Observed**: “-“ stated change did not come true, “+” stated change materialized, “|” ambiguous, “na” not applicable. **Desirability**: “--" no-one held desirable, "-" more than two thirds held undesirable, "+" more than 2/3 held desirable, "++" everyone held desirable, “na” not applicable.  **Source: d** Maria Heiskanen (Finnish Institute for Health and Welfare) personal communication December 2021 [38]

The integration of drug-related health services into mental health services was accurately predicted: more than two-thirds of the experts agreed with the statement “There are notably more combined mental health and drug addiction care units in municipalities in 2020”. Although data on this issue are not entirely comparable, the proportion of combined units seems to have more than doubled during the period (Table 5).

The developments reflecting the statement “Access to mental health care, including medication, has improved considerably in 2020, and this has diminished drug use” – which the experts did not agree with – were ambiguous. Unfortunately, no studies on care accessibility have been conducted or at least have not been published. However, we do know that drug use has increased (Table 1).

As the experts foresaw, no measures were taken in the direction that “Harm reduction has led to implementing drug consumption rooms” (Table 5). Initiatives toward this direction were made, but they did not gain momentum in the parliament [25]. A citizens’ initiative was made in February 2022 on the issue and gathered the required 50,000 + signatures [39] and entered the parliamentary process in 2023. The statement “A substitution therapy has been implemented widely for amphetamine addiction” received ambiguous evaluations in the years 2009–2011, with some experts hoping that ongoing drug trials in Finland and New Zealand would result in a therapeutic breakthrough. Unfortunately, a meta-analysis by Bhatt and others [40] showed no efficacy of psychostimulant medication for amphetamine use disorders. However, there is low-strength evidence that methylphenidate may reduce amphetamine use [41] and the latest observational studies encourage randomized clinical trials on lisdexamphetamine among persons with amphetamine dependence [42].

The prediction “People who use drugs intravenously can be forced involuntarily to therapy in 2020” was not deemed likely to materialize, and no such changes in legislation have been implemented as of 2023. Likewise, no legislation mandating that “Underaged drug users can be forced involuntarily to therapy in 2020” has been initiated; however, underaged drug users can be taken into custody if it is deemed that the use of drugs seriously endangers her or his health or development [43].

### Attitudes toward drugs

Two statements on attitudes were evaluated: “Drug use approval has widened in sports, business and cultural circles” and “The society is markedly more negative toward drugs and the consequences are more severe than now”. The first statement was predicted to materialize, but the experts were undecided on the second. Attitudes have been measured in population surveys in 2010, 2014 and 2018; however, analyses cannot be made based on, e.g., profession. The proportions fully or partially agreeing with the claim that “drugs can be used in a reasonable way that does not cause problems” increased throughout the 2010s (Table 6). The proportion believing in the possibility of nonproblematic drug use was common among those aged 25 to 34 years (Table 6). In 2019, a Citizens’ initiative proposing depenalization of cannabis use gained enough public support to proceed for consideration in the Parliament [44]. Population surveys show no indication of a more negative general attitude toward drugs, and the consequences of being found to use drugs have not become more severe. Instead, public debate on the consequences of labeling drug experimenters has risen [45].

**Table 6:**
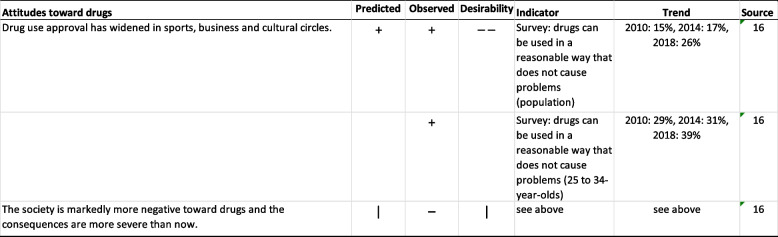
Predicted changes in attitudes toward drugs from 2009-2011 to 2020

**Predicted**: "--" no-one agreed, "-" more than two thirds disagreed, "+" more than 2/3 agreed, "++" everyone agreed. **Observed**: “-“ stated change did not come true, “+” stated change materialized, “|” ambiguous, “na” not applicable. **Desirability**: “--" no-one held desirable, "-" more than two thirds held undesirable, "+" more than 2/3 held desirable, "++" everyone held desirable, “na” not applicable

## Discussion

Regarding drug foresight, the first question is inevitably related to the extent of drug use: will the use of illicit drugs increase or decrease? In 2009–2011, the majority (but not all) of experts predicted that the use of drugs would be more common in 2020 than it was ten years earlier. They were correct: despite measures to control the use and trade of drugs, more people experimented with or used drugs, and more drug residues were found in wastewater monitoring. Population approval of the use of drugs was correctly predicted to increase, but this development has been rather slow. The substance profile did not change considerably: contrary to predictions, the use of new synthetic drugs did not increase markedly. However, the misuse of buprenorphine increased during the 2010s. In line with predictions, the incidence of polydrug use did not decrease.

In the drug market, unit prices have been surprisingly stable over the ten-year period. This phenomenon may or may not relate to the accurately predicted increase in the role of organized crime during the past decade. In the domain of drug control, the legal status of drugs did not change, in line with the predictions of the experts. However, law enforcement seems to have shifted toward more lenient forms of punishment, such as giving more notices instead of fines. In addition, the prison service started a project in 2021 [35] to place prisoners with short prison sentences and substance abuse problems in an out-of-prison substance abuse treatment facility. It seems that while legal changes have not advanced, enforcement has moved in the direction foreseen by experts ten years ago.

Drug care system reform favoring combined mental health and addiction care units took place between 2010 and 2020, as foreseen by the experts. In 2009–2011, the experts were unanimous in seeing a development toward drug therapy being governed within primary care, while in fact, the role of specialized care has strengthened. Unfortunately, no data exist on how this has affected access to care. A unanimous view that drug consumption rooms would not be implemented before 2020 was correct; however, stronger initiatives toward this direction have been made around and after 2020. Involuntary care has not been expanded in drug care, as was predicted in 2009–2011.

### Factors affecting foresight accuracy

What can be said about which aspects the experts got right and which wrong? It seems to have been easier for the experts to foresee the continuation of existing trends, e.g., increasing use of drugs or widening approval of drugs, than it was to predict possible changes in the popularity of distinct groups of drugs such as NPS or misuse of psychoactive medicine. Another dividing aspect was the extent of influence the decision-makers had on the issue under consideration. Rather surprisingly, the addiction care system moved in the opposite direction than the drug experts predicted and worked toward more specialized care instead of an increasing role of primary care. It remains to be seen if the extensive care system reform started in Finland in the beginning of 2023 [46] can change this development; the plans from 2009 on [47, 48] have aimed at increasing the role of primary care.

On the other hand, even armed with the prediction that drug import and wholesale would increasingly fall into the domain of organized crime, this undesirable development could not be stopped. However, this was something that the experts seem to have been aware of: the increasing power of organized crime was acknowledged as unavoidable [14]. Law and the implementation of law were clearly difficult issues for the experts when the predictions were made in 2009–2011: disagreement on the future direction was widespread, as was the desirability of different reforms.

When the drugs foresight 2020 study was initiated in 2009, a close look at the then recent UK Drugs Futures 2025 [5] seemed to predict a future that was notably, even dramatically, different from the situation at the time when the foresight exercise was made. For example, the role of genomics in informing individuals which ‘recreational’ drugs to use and how to use them was foreseen [5]. The Finnish Drugs Foresight 2020 study set the timeframe at ten years instead of the British study’s 20 years. A ten-year timeframe was used in a Swedish study [49], which came out with largely similar predictions as the Finnish study [6]. This decision seems to have been beneficial in framing foresight in a more down-to-earth way of thought, resulting in a more accurate view of the future. One obvious example was the effect of population aging on drug use prevalence: the experts seem to have realized that population aging is an extremely slow process with no observable influence within a ten-year timeframe.

Expert predictions and their accuracy have been a growing research interest. Some studies have concluded that scientists’ performance is no better than lay peoples’ or that of simple statistical methods [50] while others display superior experts’ performance [51]. A higher number of experts has been shown to increase accuracy as well as experts having had scientific expertise in the prediction domain [52–55]. The number of experts in the current study was small and had, to our knowledge, no training in forecasting. In light of these shortcomings, it can be noted that the foresight exercise was surprisingly successful and encourages further development. The study also revealed important issues requiring attention when planning future studies (see *Methodological issues*).

### Foresight as a tool to create a desirable future

Can foresight be utilized to alter the future in a desired direction? The foresight literature often reminds us that foresight is not so much about predicting the future but rather a tool to shape the future in a desired direction [5, 13]. The majority, but not all, of the experts thought that it was undesirable to see a future where the misuse of prescription drugs would be more common than the use of illegal substances (Table 1). After the foresight study was published, although hardly as a direct result, increased efforts were made to alter professional practices in prescribing benzodiazepines. The prevalence of long-term benzodiazepine use [56] declined significantly during the 2010s in Finland [57]. In 2020, opioid abuse in Finland consisted almost only of nonmedical use of opioid pharmaceuticals, most commonly buprenorphine, which is associated with obvious advantages over using heroin.

Should we focus on issues with widespread unanimity or those with scattered views? The Delphi method promotes a shared view of the issues under consideration and encourages consensus [58, 59]. The role of Delphi as an expert ‘discussion forum’ has strengthened since the spread of real-time Delphi [15] utilizing web-based Delphi tools. In the Delphi study in 2009–2011, experts unanimously predicted that drug therapy would be integrated into primary care – furthermore, they unanimously stated that this was a desirable direction. Therefore, it remains unclear why the proportion of drug-related interactions in specialized care increased. One possible explanation would be the question of power. Experts in drug issues may have limited influence on wider resource allocation questions involving the entire health care sector; specifically, in Finland, specialized care funding has increased more rapidly than funding for primary care [60]. Additionally, reaching a political agreement on a large-scale care system reform took almost two decades before finally taking effect in the beginning of 2023 [46].

Not all questions can be agreed upon, however, and expert disagreement can also be seen as a valuable indication of the uncertainty regarding the future. In the original study in 2009–2011, disagreement among the experts was widespread with respect to the future of drug control (Table 3). Not only did the experts disagree on the probable future – they also disagreed on the desirability of different legal reforms. The experts participated in the Delphi study anonymously: no data were gathered on the respondents’ professional backgrounds. Invitations to participate were sent to all members of the EMCDDA Reitox network national experts [6], a group including people from health care institutions as well as internal security institutions. Regarding the drug control situation in 2020, reforms have either not taken place at all or have been gradual shifts in implementation (Table 3).

The Drugs 2020 foresight study was not followed up with an advocacy plan. The results were shared with all participants of the Delphi study and published as a monograph in the Police College of Finland research series [14]. Importantly, not only were the predictions published but also the verbal justifications attributing to the views of the future. However, we have no knowledge of the extent to which this information was utilized by the experts in their corresponding agencies.

### Methodological issues

The informants in the original Delphi study were from an established national network of experts brought together for European Union drug situation monitoring purposes. However, experts not belonging to the national network, such as university researchers, clinicians or people who use drugs were not included in the Delphi study. A different set of experts might have come up with different views of the future. Future studies should consider formulating predictions that can be scored for accuracy (e.g. [52, 53, 55]). Some wordings in the original Delphi study in 2009–2011 included suggestions as to why a certain change might take place. This complicates their usability in assessing the accuracy of these predictions – if a suggestion needs to be made, it would be advisable to do it in a separate guiding text. The current study aimed to assess the accuracy of the expert predictions, but no monitoring mechanisms were put into place in 2009–2011 for this purpose. Thus, the evaluation was based on information originally gathered for other purposes. However, a thorough effort has been made to utilize as wide an information base as possible, including the use of official statistics, surveys, health care register data, wastewater monitoring data and administrative documents. Some important information is lacking, such as developments in access to drug therapies. Nonetheless, although the assessment of some individual questions may have been inaccurate, the coverage of information sources has been substantial. In issues where multiple information sources have been available, the picture has been coherent: the sources have confirmed one another.

## Conclusions

The use of foresight techniques to illuminate probable future developments in the drug field has expanded since pioneering work in the first decade of the millennium [13, 49]. In terms of drug-related issues, the results suggest that foresight can produce relatively accurate and realistic views of the future at least up to ten years ahead. For the purposes of developing health care as well as legislation, this should be sufficient. In a perfect world, the results of a foresight exercise should guide actions to achieve a desired future. Important issues to remember are the unintended and unforeseen effects that the dissemination of anticipatory statements may have. Also, it would be beneficial to set up instruments to monitor the development in the issues deemed to be of greatest importance.

## Data Availability

All data analyzed during this study are included in this published article through links in the References section.

## Abbreviations

NPS: New Psychoactive Substances
STI: Science, Technology and Innovation
UK: United Kingdom
EMCDDA: European Monitoring Centre for Drugs and Drug Addiction
THL: Finnish Institute for Health and Welfare
NBI: National Bureau of Investigation; Police of Finland
HIV: Human immunodeficiency viruses
